# Políticas públicas adoptadas en la pandemia de la COVID-19 en tres países de América Latina: contribuciones de la Promoción de la Salud para no volver al mundo que existía

**DOI:** 10.1177/1757975920977837

**Published:** 2020-12-20

**Authors:** Júlia Nogueira, Dais Gonçalves Rocha, Marco Akerman

**Affiliations:** 1 Universidade de Brasilia, Brasilia, Brasil; 2 Universidade de São Paulo, São Paulo, Brasil

**Keywords:** políticas públicas, COVID-19, América Latina

## Abstract

El mundo, después de la pandemia de la COVID-19, no será el mismo que existía y ni tan siquiera eso es deseable. La reanudación de la "normalidad" (que solo es "normal" para unos pocos) sería el regreso a la producción y el consumo insostenibles, la pérdida de derechos, la exacerbación de las iniquidades y una brutalidad diaria para muchos que luchan por sobrevivir. En América Latina (AL), el fin del mundo ya se anunció incluso antes de la pandemia. Si, desde Bogotá - 1992, la Declaración de Promoción de la Salud denuncia la pobreza como el mayor determinante social de enfermedades en la región, los gobiernos parecen ignorarla, debilitando los sistemas de salud, educación, ciencia y tecnología, transferencia de ingresos y asistencia social bajo los auspicios de la austeridad fiscal, favoreciendo el aumento de la desigualdad. Usando datos de tres países, Argentina, Brasil y Chile: 1) se señalan sus bajas inmunidades sociales anteriores a la pandemia; 2) se analizan sus políticas públicas en respuesta a la pandemia, considerando los diferentes enfoques de intervención en salud: grupos de riesgo, población, vulnerabilidad y proporcionalismo universal; y, 3) se indica cómo la Promoción de la Salud (PS) podría influir sobre las políticas públicas para no volver al mundo que existía, usando como ancla dos documentos lanzados durante la pandemia y que explicitan la lente epistémica de la comunidad global de la PS en cinco puntos: intersectorialidad, sostenibilidad, empoderamiento, compromiso con la salud pública y equidad, y perspectiva de curso de vida. Evidencias producidas en el transcurso de la pandemia en los tres países indican que los enfoques adoptados en las políticas públicas pueden, o no, favorecer el alcance de la agenda inconclusa de la PS y del desarrollo sostenible.

## Introducción

En América Latina (AL), la elevada inequidad exige mejoras en los niveles de salud en los grupos económicos más desfavorecidos. Para que las políticas públicas en el área de la salud tengan el objetivo, no solo de promover la salud poblacional, sino de reducir las inequidades, ellas tendrían que estar fuertemente integradas con otras políticas de protección social (1,2).

La pandemia de la COVID-19 llega en un momento en el que la inmunidad social de AL ya está baja. Los sistemas de salud, educación, ciencia y tecnología, transferencia de renta y asistencia social han sido sistemáticamente debilitados en la región bajo la austeridad fiscal, ampliando las asimetrías entre las poblaciones y la pérdida de derechos (3,4).

Los movimientos sociales fueron criminalizados y debilitados y el neoliberalismo se agudizó resultando en lo que Achille Mbembe denomina necropolítica, una lógica que siempre ha estado en el corazón del neoliberalismo y que opera con la idea de que unos valen más que otros y quien no tiene valor puede ser descartado (5). En contraposición a la necropolítica y para que haya una AL justa y saludable, debemos buscar “una empatía radical (6)”.

El 12 de julio de 2020, fecha de conclusión de este artículo, el mundo presenta 12.925.331 personas infectadas por el virus Sars-Cov2 y 569.097 muertes (7). Como resultado, sistemas de salud sobrecargados y graves impactos económicos y sociales. En un efecto en cascada, las paralizaciones causadas por la pandemia acarrean el endeudamiento de los países y pérdidas de empleo, ingresos y salud de los individuos (8).

AL enfrenta la pandemia desde una posición más débil que la de otros países más desarrollados y el impacto final de esa crisis dependerá de las medidas socioeconómicas y sanitarias que se tomen en el ámbito regional, nacional y mundial (9). Después de siete años de crecimiento lento, la región podrá tener el mayor desplome del PIB en un siglo (–5,3%), llegando a 83,4 millones de personas en extrema pobreza en 2020 (8).

Considerando la interrelación entre salud y economía, política, medio ambiente y cultura, se señala la falacia de la narrativa que dicotomiza la solución como salud o economía. La respuesta a la crisis sanitaria pasa por acciones específicas en la salud pública, pero también por evitar el colapso económico (8). Los diferentes abordajes de intervención adoptados por los países pueden modelar social, política y económicamente la región.

A partir de datos federales disponibles en Internet compilados por la Comisión Económica para América Latina (CEPAL), tres países son elegidos por sus impactos políticos y económicos en la región: Argentina, Brasil y Chile. Anclados en el paradigma de la determinación social de la salud (10), los investigadores realizan un ejercicio analítico para problematizar en qué puede contribuir la lente de la Promoción de la Salud (PS) para no volver al mundo que existía anteriormente.

En primer lugar, se demuestran las bajas inmunidades sociales resultantes de las políticas públicas que se venían adoptando en el contexto pre-pandemia. A partir de eso, se analizan las respuestas federales durante la vigencia de la pandemia, usando los diferentes enfoques de intervención en salud: grupos de riesgo (11), población (12), vulnerabilidad (13) y proporcionalismo universal (14).

Finalmente, se indica cómo la PS podría influir sobre las políticas de protección social, sanitaria y económica para no volver al mundo que existía. Para ello, se utilizan como ancla dos documentos lanzados durante la vigencia de la pandemia (1,2) y que explicitan la lente epistémica de la comunidad global de la PS en cinco puntos: intersectorialidad, sostenibilidad, empoderamiento, compromiso con la salud pública y equidad y perspectiva de curso de vida.

### El mundo que existía: contexto de tres países de América Latina

Un mundo que optó por un modelo injusto e insostenible de desarrollo provocó esta situación crítica. En los últimos 50 años, los adeptos del neoliberalismo, al mismo tiempo que niegan las crisis socioeconómica y climática producidas por ese modelo, tratan de escapar de sus consecuencias construyendo bastiones inaccesibles a una multitud que fue dejada atrás, sin la división universal de los “frutos del progreso (15)”.

Al reconocer la determinación social de la salud, en la Tabla 1, se presentan datos para una mirada comparativa entre los países, además de una síntesis panorámica de las políticas públicas.

**Tabla 1.** Características socioeconómicas y sanitarias de Brasil, Argentina y Chile.

Características	Países		
	Brasil	Argentina	Chile
Población ^a^	212.413.908	45.155.378	19.100.270
PIB (per cápita US$) (BM, 2018) ^b^	9.001,2	11.683,9	15.923,4
Gasto público en salud (per cápita US$) (BM, 2017) ^c^	616,56	1.388,16	1.115,58
IDH (PNUD, 2018) ^d^	0,761	0,830	0,847
Esperanza de vida al nacer (años) (PNUD, 2018) ^d^	75,7	76,5	80,0
Mortalidad infantil (IGME 2018) ^e^	12,8%	8,8%	6,2%
Mortalidad COVID-19 / 1M pop por 100 mil ^a^	336	40	360

Elaboración propia a partir de datos disponibles en:

a https://www.worldometers.info/coronavirus/;

b https://data.worldbank.org/indicator/NY.GDP.PCAP.CD;

c https://data.worldbank.org/indicator/SH.XPD.CHEX.GD.ZS;

d http://hdr.undp.org/sites/default/files/hdr_2019_pt.pdf;

e https://estadisticas.cepal.org/cepalstat/perfilesNacionales.html?idioma=spanish

En AL, el mayor reto de los gobiernos debería ser disminuir las desigualdades socioeconómicas que impiden un desarrollo más equitativo, integral e inclusivo. Entre los países, los sistemas de salud, aunque distintos en su estructura y modelo de financiación (16–18), atienden a una población vulnerable, en envejecimiento y con un perfil epidemiológico donde predominaban las enfermedades no transmisibles (19).

#### Brasil: austeridad y regresividad sin precedentes en los derechos

Desde 2016, Brasil enfrenta una crisis político-económica sin precedentes. Para reducir el déficit fiscal, una Enmienda Constitucional congeló los gastos primarios del gobierno federal durante un período de 20 años sin considerar medidas capaces de reducir también la desigualdad, como la lucha contra la evasión fiscal o una reforma tributaria progresiva (20). En 10 años, esa reforma debe reducir el gasto primario del 20% al 12% del PIB, afectando principalmente el presupuesto para infraestructura y programas sociales, causando reveses en los “derechos de la ciudadanía”. Cortes en programas de transferencia de renta, de enfrentamiento de la violencia de género y en el Programa de Adquisición de Alimentos amenazan anular los avances en la seguridad alimentaria que habían hecho que Brasil saliera del mapa del hambre (21). A su vez, la Reforma de la Previsión Social desmantela el principal mecanismo redistributivo y de protección social del país (22). Tales medidas mantienen privilegios fiscales para los más ricos, haciendo que sea inviable el alcance de los objetivos de desarrollo sostenible (ODS) (23).

#### Argentina: subejecución de los gastos sociales y violación de la transparencia fiscal

Desde 2015, el Estado argentino ha disminuido los instrumentos de protección social y el derecho a la seguridad social, fallando en la implementación de políticas fiscales tendientes a la igualdad. En 2017, realizó una interrupción sin previo aviso – incumpliendo así los estándares vigentes en el debido proceso administrativo – y masiva de pensiones no contributivas de personas con discapacidad (24). Además, el gasto social para garantizar los derechos en la infancia disminuye y se realiza de manera ineficiente, provocando desigualdades (25). La débil capacidad de intervención del Estado en los procesos de desarrollo territorial y la disminución del impuesto inmobiliario, que hoy representa menos del 1% de la carga tributaria total, desfavorece la redistribución de la renta proveniente de la explotación comercial del suelo e incentiva la iniquidad (26).

#### Chile: aparente éxito neoliberal que camufla las desigualdades sociales

Chile presenta resultados positivos en indicadores de desarrollo económico y de salud global que lo sitúan como uno de los países con mejor índice de capital humano en AL (27). Sin embargo, la propuesta de desestatización, nacida con la justificativa de auxiliar en el crecimiento económico, y de privatización de la Previsión Social, en la década de los 80, enfrenta uno de sus momentos más complejos. La reducción en el valor de las pensiones y jubilaciones causa una creciente ola de suicidios entre los ancianos (28). Desde 2019, hay una convulsión social con múltiples y permanentes manifestaciones de descontento. El gobierno, obligado a proponer modificaciones al sistema de salud como seguro público, lo hace con propuestas cosméticas (17). La incidencia de la pobreza (el 8,6%) presenta diferencias según vulnerabilidades: lugar en el que se vive (del 16,5% en el sector rural al 7,4% en el urbano), pertenencia a grupos específicos (17,2%, en la región con alta concentración de pueblos indígenas), sexo y grupos de edad.

El colonialismo territorial puede haber terminado, pero esta crisis actual sirve de recordatorio de que la colonización de la salud, de la economía y de la política permanece viva (29). Durante la pandemia, naciones (en particular las de ingresos altos) cerraron sus fronteras al exterior y se enfocaron en el contexto interno. Con eso, la AL se la recuerda por las estructuras de poder asimétricas que dominan el concepto de desarrollo global y de salud. “El modelo global de salud se basa en gran parte en la asistencia técnica y en la capacitación de los países ricos, cuya respuesta a esta pandemia fue esclerótica y atrasada en la mejor de las hipótesis (30)”.

A pesar de los grandes avances en salud en los últimos 50 años, esas ganancias no beneficiaron a todos igualmente; las inequidades aumentaron (31). Reanudar las discusiones sobre los valores que rigen nuestras sociedades significa enfrentar un *statu quo* favorable a la enfermedad y (re)politizar el papel de la formación política participativa y emancipadora para la salud.

### El mundo en construcción: análisis de las medidas de protección adoptadas durante la pandemia en países de América Latina

El análisis de las políticas adoptadas en el transcurso de la pandemia de la COVID-19, fundamentado en los enfoques de intervención, permite identificar si ellas producen inclusión o exclusión, protección o estigma, vida o muerte (14,32,33). Esos abordajes corresponden a diferentes maneras de aliviar la carga injusta de las enfermedades. La tipología de política propuesta puede servir como herramienta eficaz para interpretar el impacto potencial de intervenciones en diferentes grupos.

En este punto, a partir de la información contenida en los documentos de organizaciones multilaterales como BID (3) y CEPAL (8), se analizan las políticas públicas formuladas por tres países latinoamericanos en respuesta a la pandemia en las dimensiones: medidas de contención, económicas, empleo, protección social y educación (Tabla 2).

**Tabla 2.** Brasil, Argentina y Chile: medidas de contención, protección social, económica, laboral y educativa en el enfrentamiento de la COVID-19 adoptadas entre marzo y mayo de 2020.

Medidas	Países		
Dimensiones	Brasil	Argentina	Chile
**Contención** Confinamiento y cierres de emergencia	- No hay orden nacional de confinamiento.	- Decreto nacional de confinamiento social obligatorio horizontal (acuerdo entre diferentes ministerios) con resolución adicional para ancianos.	- No hay orden de confinamiento nacional. Confinamiento por regiones con especificación adicional para ancianos (obligatorio para mayores de 80 años).
	- Recomendación de confinamiento vertical (por sector de servicio público).	- Cierre de establecimientos comerciales no esenciales (tiendas de alimentación, farmacias continúan abiertas).	- Toque de queda (22h00 a 05h00).
	- Restricción de actividades económicas a cargo de los estados.	- Escuelas y universidades cerradas.	- Restricción de desplazamiento y de algunas actividades económicas
	- Cierre de escuelas y universidades en algunos estados.	- Todas las fronteras cerradas.	- Cierre de escuelas y universidades.
Restricciones de frontera	- Cierre de las fronteras terrestres.		- Cierre de las fronteras terrestres para extranjeros no residentes.
	- Entrada permitida por vía aérea de ciudadanos brasileños y residentes.		- Cuarentena obligatoria para los ciudadanos y residentes que llegan al país.
	- Restricción al desplazamiento entre estados.		
**Económicas**	- Flexibiliza las condiciones de pago y renegociación de deudas.	- Creación de líneas de crédito para asegurar la producción y el abastecimiento de alimentos e insumos básicos y dar impulso a la actividad.	- Aplazamiento del pago del Impuestos (tres meses) para PYMES.
	- Moratoria parcial de impuestos sobre nómina de pagos (tres meses).	- Relanzamiento del Plan Procrear para dar impulso a la construcción civil.	- Adelanto del reembolso del impuesto sobre la renta para PYMES.
	- Crédito de emergencia para PYMES (Pequeñas y Medianas Empresas) y para el mantenimiento de empleos.	- Aumento de gastos de inversión en obras de infraestructura, educación y turismo.	- Reducción del impuesto de sello, 0% para transacciones de préstamos o créditos (seis meses) para empresas e individuos.
	- Reducción de la tasa de interés en 50 puntos básicos (para el 3,75%).		- Aumento de gastos de inversión.
	- Intervención en mercado cambiario		- Reducción de la tasa de interés en 50 puntos básicos (para el 0,5%).
**Protección Social**	- Amplia el programa de transferencias condicionadas.	- Transferencias extraordinarias para:	- Transferencias extraordinarias para:
	- Transferencias extraordinarias para trabajadores informales y microempresas.	personas sin ingresos y que no reciban otros subsidios;	desempleados;
	- Adelanto de sueldo a empleados con ingresos hasta dos salarios mínimos, y cuyas jornadas hayan sido reducidas.	jubilados y pensionistas;	beneficiarios del programa Subsidio Único Familiar;
	- Adelanto del aguinaldo.	beneficiarios del auxilio universal.	familias más vulnerables.
	- Línea de crédito para empresas con gastos en nómina de pagos.	- Maternidad/Embarazo.	- Flexibiliza pagos de deudas de personas de bajos ingresos (sin intereses ni multas).
	- Flexibiliza la legislación laboral.	- Compensación salarial para trabajadores con ingresos reducidos.	- Flexibiliza la legislación laboral.
	- Autorización a líneas de créditos para distribuir préstamos a la población de bajos ingresos, PYMES y autónomos.	- Exención de las contribuciones patronales a la previsión social.	- Asegura el ingreso del trabajador (por medio del seguro desempleo) y las contribuciones previsionales (por cuenta del empleador).
		- Control de precios de bienes básicos.	- Permite reducir la jornada de trabajo, compensando con recursos del Fondo de Desempleo Solidario.
		- Prohibición de suspensión de servicios básicos por falta de pago.	- Adelanta pagos a proveedores del Estado.
		- Flexibiliza la legislación laboral.	
		- Subsidio y financiación para asegurar el teletrabajo de empleados.	
**Empleo** Teletrabajo	- Habilitado para grupos de riesgo.	- Obligatorio para actividades no esenciales.	- Incentivado para grupos de riesgo.
Licencia y Seguro de desempleo	- Prevista por ley laboral.	- Prevista por ley laboral.	- Garantizado para la COVID-19.
	- Programa para protección de empleo e ingreso (para empresas).	- Ampliación de medidas existentes de forma general y específicas.	
**Educación** Suspensión	- Restricción de actividades a cargo de los estados.	- Acordado entre gabinetes ministeriales, especialistas y estados.	- Suspensión en todos los niveles.
Enseñanza a distancia	- Autoriza funcionamiento en línea sin recursos.	- Lanza plataforma educativa; radio, TV y paquete de datos.	- Plataforma en línea gratis (hasta la secundaria).
Alimentación escolar	- Permite distribución de alimentos con recursos de alimentación escolar.		- Distribuye canastas a todos los alumnos que recibían alimentación en la escuela.

Fuente: Adaptación de los datos compilados en https://www.cepal.org/pt-br/taxonomy/term/834.

Graham (32) identifica tres tipos de abordajes políticos reflejando valores y objetivos diferentes: 1) Población de Riesgo, se enfoca en la reducción de la exposición al riesgo específico para individuos por medio de programas direccionados y cambios comportamentales, para mejorar la salud de las personas en el peor escenario, sin considerar aquellos que están en mejor situación; 2) Poblaciones Vulnerables, busca atenuar las lagunas de salud o estrechar la separación entre aquellos que están en las peores situaciones y los mejores grupos; y 3) Poblacional, que por medio de cambios en las condiciones ambientales que llevarían al aumento del riesgo, invierte en el desplazamiento de la distribución de la población expuesta a un riesgo menor y reduce las desigualdades sociales.

En el actual contexto de la COVID-19, el abordaje en subpoblaciones específicas con enfoque en grupos de riesgo puede representarse por las medidas de confinamiento selectivo de ancianos y portadores de comorbilidades. Este ha sido el abordaje defendido por el gobierno brasileño y ampliamente criticado, puesto que no considera las barreras sociales y económicas para el confinamiento y no eleva los niveles de salud en los grupos más vulnerables (33). Los progresos en salud entre grupos en mejor situación pueden llevar al aumento de las inequidades. Además, estigmatiza grupos (peligrosos, contagiosos) y mina la política de solidaridad que es la clave para mantener el apoyo con recursos públicos.

Una política puede no encajarse necesariamente en un único enfoque. Las estrategias direccionales y universales no son mutuamente excluyentes, sino que pueden ser complementarias y desarrollarse. Chile realizó una combinación de estos abordajes, alternando confinamiento total y por regiones, considerando predominantemente el riesgo biológico y no las vulnerabilidades sociales. Las inequidades socioespaciales y condiciones de vivienda, y utilización de las tropas militares para el toque de queda nocturno enfatizan la intervención con baja participación social en la toma de decisiones.

Un cuarto tipo de categoría de intervención: el Universalismo Proporcional, o política universal con beneficios por medio del gradiente, enfoca los problemas o determinantes de la salud, cuya ocurrencia aumenta con la desventaja social (14). Las acciones son universales, pero con escala e intensidad proporcionales al nivel de desventaja, lo que disminuye el hiato entre los grupos, pero todos mejoran.

Este abordaje fue adoptado por el gobierno argentino que decretó el confinamiento social obligatorio horizontal en el ámbito nacional (pactado con los diferentes ministerios de forma intersectorial) y con resolución adicional de transferencia de renta para pensionistas y beneficiarios de programas sociales. También, mediante articulación interinstitucional e intersectorial, adoptó medidas para el enfrentamiento de las situaciones de violencia de género, incluyendo acciones fiscales del Ministerio Público.

El abordaje dirigido al estrechamiento de las “lagunas en la salud” busca la reducción de la diferencia entre los grupos más ricos y los más pobres (14). Ese abordaje busca revertir tendencias y alinea políticas de equidad en salud con políticas más amplias. No obstante, continúa dirigiendo esfuerzos para la subpoblación en peor situación, medida en relación con la mejor, pudiendo fallar en la mejora de la salud de grupos intermediarios en el gradiente socioeconómico.

### Contribuciones de la Promoción de la Salud para no volver al mundo que existía

La Tabla 2 muestra que hay respuestas gubernamentales de protección social, sanitaria y económica que son diferenciadas y con niveles de efectividad social variadas. ¿Las referencias recientes de la PS (1,2) pueden servir como lentes /inspiración para modelar social y políticamente la AL que se desea durante las pandemias o en los períodos entre ellas?

El propósito de promover la salud envuelve el fortalecimiento de la capacidad individual y colectiva en la solución de problemas que afectan los modos de vivir y de enfermarse de sujetos en sus territorios. Eso se fortalece al combinarse con los aspectos: confianza, solidaridad, apoyos mutuos, alianzas, acción intersectorial, movilización social, compromiso, capacidad de resistencia (resiliencia), protección a los más frágiles, acceso a fondos, habilidades digitales, entre otros. Ese conjunto de aspectos podría agruparse y denominarse potencias de un territorio social para la producción de salud y vida.

Amparados en 34 años de acumulación de la comunidad epistémica global del campo de la PS (1), se presentan cinco puntos de discusión que permiten la reflexión sobre aspectos promocionales de la vida y salud:

1. Intersectorialidad: moviliza la acción colaborativa entre sectores de gobierno y entidades de la sociedad civil.
2. Sostenibilidad: fortalece la capacidad de reacción de los servicios de salud pública y de asistencia sociales; con prácticas que transformen la competencia y fortalezcan la solidaridad; sin descuidar el equilibrio ambiental.
3. Empoderamiento y Participación en las medidas de salud pública: por medio de información válida y adecuada (culturalmente apropiada y que aborde los aspectos de interfaz con la COVID), no solo en términos de protección individual, sino principalmente en el aumento de la resistencia colectiva.
4. Equidad: como “no están todos en el mismo barco” (34) hay que observar si los diferentes grupos sociales están siendo tratados de forma diferente.
5. Perspectiva de ciclos de vida: reconocen las diferencias de riesgos entre niños, jóvenes, mujeres, trabajadores, ancianos y propicia medidas diferenciadas para cada grupo en sus territorios.

En un modelo ideal, no se volvería al mundo que existía, con sus defectos, si una articulación estratégica de esos cinco puntos se consustanciase en un ejercicio de gobernanza sólida y transparente y que generara confianza y apoyo por parte de los ciudadanos. Eso se traduciría en efectiva colaboración entre niveles de gobiernos (intra e intersectorialmente) y los movimientos sociales para producir compromiso y participación social en la formulación, implementación, control y evaluación de las políticas públicas.

Tales mecanismos de articulación y participación podrían fortalecer la sostenibilidad de las políticas y del desarrollo humano, siempre y cuando todos estuvieran imbuidos por un profundo compromiso ético-político con la equidad, significando que las políticas públicas harían sus asignaciones de recursos y servicios llevando en consideración las distintas singularidades y necesidades de los sujetos en cuestión.

¿Cómo es la utilización de esa lente de la PS en el mundo real de la pandemia y de las políticas de protección social, planes de contingencia y medidas económicas?

1. Se observan señales de desarticulación entre las esferas de gobierno, menos señalada en Argentina y Chile, países con mayor centralización federal, y más señalada en Brasil, donde hay mayor autonomía garantizada por la Constitución a los entes de estados y municipios.
2. Se captan grados de solidaridad con los trabajadores y empresarios en la flexibilización de la legislación laboral y del pago de impuestos, señaladamente más generosos en Argentina que en Brasil o en Chile y se reconoce la ausencia de preocupación por parte de los gobiernos por el equilibrio ambiental.
3. Se ven acciones dirigidas al aumento del empoderamiento colectivo, más señaladas en Argentina, donde las políticas parecen crear posibilidades de apoyo mutuo entre los ciudadanos, favoreciendo un mayor compromiso y priorizando la inversión social, mientras que en Brasil y Chile la legislación parece priorizar la protección individual, responsabilizando a las personas por sus cuidados.
4. La pandemia desnuda las inequidades sociales en los tres países. Sin embargo, se observan medidas de emergencia de protección para asegurar la supervivencia de los más frágiles que, no obstante, no alteran la estructura de distribución de recursos, servicios y poder.
5. Las políticas argentinas explicitan la perspectiva de ciclo de vida, dando apoyo a jubilados y pensionistas, embarazadas, estudiantes y trabajadores en sectores más afectados. Chile mira con atención a los estudiantes. Brasil no adopta la perspectiva de ciclo de vida en sus políticas.

Las estadísticas actuales de la pandemia en los tres países parecen reflejar la efectividad de las políticas. Argentina, que priorizó la articulación, incluso entre fuerzas políticas diferentes; mayor solidaridad; legislaciones que favorecen la resistencia colectiva y la perspectiva de ciclos de vida en la asignación de recursos, se ve recompensada por un coeficiente de mortalidad casi diez veces inferior a los de Brasil y Chile (7).

Para enfrentar este escenario es necesario implantar una infraestructura global que permita identificar y responder a los problemas, incluso antes de que ellos asuman proporciones epidémicas. Desde la Conferencia de Bogotá, en 1992, (35) se sabe que la pobreza es el mayor determinante social de la enfermedad, que la principal amenaza a la salud es la inequidad y que la austeridad en las políticas mata (36).

Las grandes corporaciones ya empiezan a moverse para garantizar el control de lo que vendrá (37). El futuro está en disputa (38). Por medio de las políticas públicas, le cabe a la sociedad señalizar y al Estado interceder en esa disputa, dando apoyo a las luchas colectivas alrededor de proyectos que lleven a la autonomía, la solidaridad, la justicia y la equidad.

La pandemia de la COVID-19 pone en evidencia la necesidad de un compromiso intensificado y sostenido con la preparación para la salud pública global e indica la necesidad de políticas y estrategias integradas de salud orientadas hacia la equidad. Es oportuno rescatar la agenda por concluir de la PS y sus relaciones con recientes agendas globales: ODS y su lema de no dejar a nadie atrás; sistemas universales de salud y salud en todas las políticas; renta básica de ciudadanía; gravamen fiscal progresivo y sobre las grandes fortunas; organismos internacionales, políticas multilaterales y decisiones globales; inclusión digital, internet libre y educación pública y de calidad; movilidad urbana segura y ciudadana; entre otras. Invertir en salud debe pensarse como una inversión inteligente, puesto que continuará habiendo problemas como ese, particularmente en un mundo globalizado.

## Consideraciones finales

La pandemia de la COVID-19 representa un gran desafío para la salud pública con graves impactos económicos y sociales. Situaciones de austeridad y pandemia generan disminución de ingresos, pesimismo, reclusión, soledad, violencia, enfermedad y muerte. Para contraponerse a eso es necesario buscar formas de envolver a las personas para que se apoyen mutuamente.

Los gobiernos deberían garantizar sistemas de protección para aumentar la inmunidad social y la resiliencia. Como parte indispensable de una respuesta política coordinada para la crisis, las personas precisan contar con acceso a los cuidados de salud, seguridad de empleo e ingresos y educación, particularmente entre los más vulnerables, pero sin ignorar al resto de la población. Esas medidas contribuyen a evitar la pobreza, el desempleo y la informalidad y son poderosos estabilizadores económicos que pueden contribuir a una rápida recuperación.

Los enfoques de intervención, que no consideraron las causas estructurales de las inequidades en salud para el enfrentamiento de la COVID-19 y que priorizaron el enfoque en grupos específicos, con énfasis en el riesgo biológico, reprodujeron discursos y prácticas excluyentes y ampliaron el coeficiente de mortalidad. Como el objetivo de mejorar la salud de la población puede no ser necesariamente compatible con el objetivo de reducir las disparidades, las estrategias de salud pública deben incluir abordajes poblacionales universales, considerando los gradientes sociales con vistas a la promoción de la equidad.

Entre las lecciones aprendidas se destacan: la gobernanza intersectorial por medio de acciones coordinadas que favorecen la protección de la vida señala la reducción de daños de la pandemia; la necesidad de adoptar enfoques multiescala considerando la articulación de las dimensiones micro, media y macro para el enfrentamiento de las causas estructurales de las inequidades en salud; y la necesidad de avanzar en el reconocimiento de la crisis climática y de incluir la dimensión ambiental en las acciones emprendidas.

La adopción de políticas de PS salva vidas y puede entenderse como una “empatía radical” en la formulación de políticas públicas (6) que favorezca que no se vuelva más al mundo que existía. Ya no se trata más de reanudar o transformar un sistema de producción, sino de abandonar la producción como el único principio de relación con el mundo. Después de cien años de un socialismo que se limitó a pensar en la redistribución de los beneficios de la economía, tal vez sea el momento de inventar un socialismo que se contraponga a la propia producción. Porque la injusticia no se limita a la redistribución de los frutos del progreso, sino a la propia manera de hacer que el planeta produzca frutos (39).
